# Urban regrets (Unhappy metros: Satisfaction with life scale (SWLS))

**DOI:** 10.1016/j.heliyon.2024.e30729

**Published:** 2024-05-13

**Authors:** Adam Okulicz-Kozaryn

**Affiliations:** Rutgers - Camden, United States of America

**Keywords:** Urban-rural happiness gradient, Urban, Cities, Happiness, Life satisfaction, Subjective WellBeing (SWB), Satisfaction with life scale (SWLS), Panel study of income dynamics (PSID)

## Abstract

This is the first study of urban-rural happiness gradient using multi-item Satisfaction With Life Scale (SWLS). A new finding is that urbanites fail especially on “If I could live my life over, I would change almost nothing”–urban way of life tends to result in regrets. Effect sizes of urbanicity on subjective wellbeing (SWB) are substantial–about half of health–living in a metro depresses one's happiness as much as going half way from fair health to poor health, for instance.

## 1 Introduction

The objective and rationale of the study are to use a multi-item scale to study urban-rural happiness gradient. The scope is the US in 2015/2016 using quantitative survey data analysis with regression.

The urban-rural happiness gradient means that happiness raises from its lowest in largest cities to highest in smallest places, little towns, villages, and open country. Urban unhappiness is common [48], [56], [23], [31], [33]. As a corollary, exposure to nature, the opposite of urbanicity, is related to happiness [51], [22], [59], [27], [1].

There are around a hundred studies on urban-rural happiness gradient [48], [42], but they all use a simple single-item measurement of SWB. Such limitation is understandable and apparently insurmountable, as multi-item scale measurement is typically restricted to small-sample laboratory settings. And urbanicity deriving from place of residence by definition requires wide geographical coverage and large sample. This is the first study of urban-rural happiness gradient using multi-item scale measurement of SWB. We hypothesize:

*Metros are less happy across multiple measures of subjective wellbeing (SWB).*

## 2 Data

We use unique (in urban and SWB research) data, 2016 Wellbeing Module of Panel Study of Income Dynamics merged with 2015 family file (psidonline.isr.umich.edu). All wellbeing measures come from the 2016 module, and all other measures, including urbanicity come from 2015 family file. There is no corresponding 2016 family file. Such setup also helps with reverse causality–wellbeing cannot cause urbanicity (moving or staying) as it is observed afterwards. Still, as any non-experimental study, the present study cannot claim causality. We keep only the reference person (head) following Brown and Gathergood [5].

A unique advantage of PSID 2016 Wellbeing Module is multiple SWB measures. Indeed, it is the only US dataset having an extensive set of SWB measures covering both metro and non-metro areas. All variables are described in Table 1, and summary statistics are in Online Appendix. We will use several SWB measures. We start with a usual SWB item, a life satisfaction measure: “How satisfied are you with your life as a whole these days?” Next, we use a “ladder” SWB measure. And finally, we have constructed a SWLS scale using command alpha in Stata without ‘asis’ and ‘std’ options: alpha WB16A3A WB16A3B WB16A3C WB16A3D WB16A3E,gen(SWLS). Cronbach's alpha of the scale has good validity at .88. The items that have been used for the scale construction are listed under “swls items” in Table 1.

**Table 1:** Variable definitions.

name	description
**global swb measures**	
satisfied with life as a whole	“How satisfied are you with your life as a whole these days?”
life satisfaction ladder	“Suppose that the top of the ladder below represents the best possible life for you and the bottom of the ladder represents the worst possible life for you. On which step of the ladder do you feel you personally stand at the present time?”
swls	Satisfaction With Life Scale (SWLS)
**swls items**	
life is close to ideal	“How much do you agree or disagree with each of the following statements: In most ways, my life is close to my ideal.”
conditions of life excellent	“(How much do you agree or disagree with each of the following statements:) The conditions of my life are excellent.”
satisfied with life	“(How much do you agree or disagree with each of the following statements:) I am satisfied with my life.”
gotten the important things	“(How much do you agree or disagree with each of the following statements:) So far, I have gotten the important things I want in life.”
would change almost nothing	“(How much do you agree or disagree with each of the following statements:) If I could live my life over, I would change almost nothing.”
**explanatory variables**	
metro	“Metropolitan/Non-metropolitan Indicator. This indicator is derived from the 2013 Beale-Ross Rural-Urban Continuum Codes published by USDA based on matches to the FIPS state and county codes.” 1 Metropolitan area (Beale-Ross Code ER775923= 1-3) 0 Non-metropolitan area (Beale-Ross Code ER775923= 4-9)
age	age
age sq	age squared
last year total family income	last year total family income
employment status	“We would like to know about what (you/HEAD) (do/does) – (are/is) (you/HEAD) working now, looking for work, retired, keeping house, a student, or what?–FIRST MENTION”
race	“What is (your/his/her) race? (Are/Is) (you/he/she) white, black, American Indian, Alaska Native, Asian, Native Hawaiian or other Pacific Islander?–FIRST MENTION” NOTE: “latino” category derived from ER64809: “In order to get an idea of the different races and ethnic groups that participate in the study, I would like to ask you about (your/your spouse's/[HEAD]'s) background. (Are/Is) (you/he/she) Spanish, Hispanic, or Latino? That is, Mexican, Mexican American, Chicano, Puerto Rican, Cuban, or other Spanish?”
kids	“Number of Persons Now in the FU Under 18 Years of Age”
college	“Did (you/he/she) attend college?” 1='yes', 0='no'
health	“Now I have a few questions about your health. Would you say your health in general is excellent, very good, good, fair, or poor?” 1 (poor) to 5 (excellent)
male	gender
married	“Are you married, widowed, divorced, separated, or have you never been married?” 1='married'; 0 otherwhise
family unit size	Number of Persons in FU at the Time of the Interview
important to live in a city/place that one likes	“(Below is a list of things that may or may not be important to you. How important are each of the following to you:) Living in a city or place that I like.”

Diener's Satisfaction With Life Scale (SWLS) [15] consists of 5 items. SWLS is the most popular scale for measurement of life satisfaction, e.g., the original paper introducing the scale [15] is cited over 30k times.

More recently, Diener concludes that SWLS has good convergent validity with other scales and with other types of assessments of Subjective WellBeing (SWB). SWLS has some temporal stability (e.g., 0.54 for 4 years). Further, the scale has discriminant validity from emotional well-being measures [50, p. 101].

SWLS consists of 5 items. Pavot and Diener [50] argue that the fifth item is the weakest in terms of convergence with other items. This may be because four first items (especially the first three) refer primarily to the present, but the fifth item (and also possibly fourth one) refers primarily to the past. A similar point is made by Slocum-Gori et al. [57]: in terms of unidimesionality of SWLS it holds up reasonably well, except the fifth item. Oishi [38] groups together first three items as referring to external living conditions or the present level of satisfaction, and the last two items as referring to one's satisfaction with past accomplishments.

Our main explanatory variable of interest is metro dummy, a dummy variable that equals 1 if a county is metropolitan, and 0 if a county is non-metropolitan. More information about the metro classification is in Online Appendix.

We control for a usual set of SWB predictors including age, age^2^, education, gender, and marital status following Okulicz-Kozaryn and Valente [47]. Income has been possibly the most studied predictor of SWB–it predicts higher SWB but with diminishing returns or up to a point [30], [39], [58], [6], [13], [41], [17], [14], [16], [26]. It is important to control for income as it also confounds with urbanicity–incomes are higher in metros. Indeed, not controlling for income typically yields insignificant or weaker results as positive effect of income and negative of cities cancel each other out. Health is one of the strongest predictors of SWB [9].

Race is an important variable, as it not only predicts SWB, but is also confounded with urbanicity [e.g., 2]. Likewise, religiosity [40] and type of work [43] may affect SWB, and confound with urbanicity–we include additional models in Online Appendix. We also would like to control for political views as they predict SWB [44] and confound with urbanicity, but there are no political measures in PSID.

The US is a geographically diverse country with a multitude of regional differences that may affect the results, notably urban areas differ greatly depending on the region, and hence, we include state dummies.

Finally, the 2016 PSID Wellbeing Module contains an item “important to live in a city/place that one likes”–a weight that ones gives to place may affect results, hence, we include this item as a control as well.

We use ordinary least squares (OLS). Although OLS assumes cardinality of the outcome variable, and SWB measures are technically ordinal, OLS is an appropriate estimation method. Ferrer-i-Carbonell and Frijters [18] has shown that OLS results are substantially the same as those from discrete models, and OLS has become the default method in happiness research [4]. Theoretically, while there is still debate about the cardinality of SWB, there are strong arguments to treat it as a cardinal variable [34], [35], [36].

## 3 Results

**Figure 1:**
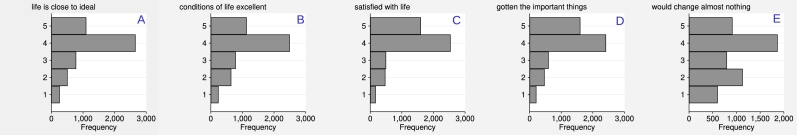
SWLS items' distribution. Panels A-E show each item.

Life satisfaction's usual distribution is left-skewed–most people are quite happy at around 6-9 on 1-10 scale. PSID SWLS items are no different as shown in Fig. 1–most people are at 4, and then at 5 and 3 on 1-5 scale. Yet the fifth SWLS item “If I could live my life over, I would change almost nothing” is slightly bimodal, still with tallest distribution at 4, but then a curious bump at 2 indicating that quite a few people do have regrets and would have changed their life if they could live again. Next we explore wellbeing measures by metro non-metro dichotomy.

In Table 2 we look at 3 global measures in first panel, and then 5 components of SWLS in second panel. There is small metro SWB penalty in 1st panel. In the 2nd panel, the first 2 SWLS components have small metro penalty as well, third component is about the same, and the last two components, especially the last one, have a substantial metro penalty. All mean differences from Table 2 will be about twice as large when controlling for full set of SWB predictors in regressions except the last SWLS item, which will be only slightly larger. This is consistent with past research–urban rural happiness gradient emerges or strengthens when controlling for predictors of SWB [48]. Unlike in Burger et al. [7].^2^

**Table 2:** Metro and non-metro means: global SWB measures in 1st panel, and SWLS components in 2nd panel.

	satisfied with life as a whole	life satisfaction ladder	swls	life is close to ideal	conditions of life excellent	satisfied with life	gotten the important things	would change almost nothing
nonmetro	3.69	7.15	3.69	3.71	3.66	3.86	3.88	3.32
metro	3.61	7.05	3.63	3.65	3.63	3.88	3.80	3.17

OLS regressions of global measures of SWB are in Table 3. Columns a1* show results from models with basic controls. While residents of metros are less happy, as expected, results are borderline statistically significant or insignificant. Addition of race categories in columns a2* raises statistical significance.^3^ Addition of evaluation whether living in a city/place that one likes is important further increases statistical significance in columns a3*. More elaborate models a3* are the “final” ones, the takeaway from the study. They suffer less from left out variable bias than initial a1* and a2* models, and are not oversaturated with less important controls (occupational sector, religiosity, and satisfaction with city) as in models c3* in online appendix. Finally, metro estimates in a3* models are very similar to more elaborate c3* models.

**Table 3:** OLS regressions of global measures of SWB.

	a1a satisfied with life as a whole	a1b life satisfaction ladder	a1c swls	a2a satisfied with life as a whole	a2b life satisfaction ladder	a2c swls	a3a satisfied with life as a whole	a3b life satisfaction ladder	a3c swls
metro	-0.08+	-0.09	-0.07+	-0.12**	-0.21*	-0.10*	-0.14***	-0.25**	-0.13**
age	-0.00	0.00	-0.02*	-0.00	-0.00	-0.02*	-0.00	-0.00	-0.02**
age sq	0.00	0.00	0.00**	0.00	0.00	0.00**	0.00	0.00	0.00***
last year total family income	0.00***	0.00***	0.00***	0.00***	0.00***	0.00***	0.00***	0.00***	0.00***
temp not working	-0.15	-0.56	-0.36	-0.17	-0.61	-0.36	-0.14	-0.55	-0.33
unemployed	-0.21**	-0.47**	-0.32***	-0.22**	-0.50**	-0.32***	-0.19*	-0.44**	-0.30***
retired	0.17***	0.19+	0.14**	0.17***	0.20+	0.14**	0.15**	0.17+	0.13**
disabled	-0.05	-0.23	-0.22**	-0.07	-0.27+	-0.23**	-0.06	-0.25+	-0.22**
housekeeping	-0.03	-0.05	-0.02	-0.04	-0.08	-0.03	-0.03	-0.07	-0.02
student	-0.18	-0.39	-0.21	-0.21	-0.46	-0.22	-0.21	-0.48	-0.24
kids	-0.07*	-0.08	-0.03	-0.06*	-0.07	-0.03	-0.06*	-0.07	-0.03
college	-0.07*	-0.20**	-0.09**	-0.04	-0.14*	-0.07*	-0.05	-0.16*	-0.08*
health	0.28***	0.56***	0.26***	0.28***	0.57***	0.26***	0.27***	0.54***	0.25***
male	-0.09*	-0.18*	-0.11**	-0.07+	-0.12	-0.10*	-0.05	-0.08	-0.08*
married	0.19***	0.51***	0.32***	0.21***	0.56***	0.33***	0.21***	0.55***	0.32***
family unit size	0.08**	0.08	0.04+	0.07**	0.05	0.04	0.07**	0.05	0.04
black				0.20***	0.52***	0.11**	0.18***	0.48***	0.09*
other				0.27+	0.45	0.12	0.27*	0.46	0.12
asian				0.11	0.16	0.10	0.14	0.22	0.13
latino				0.27***	0.72***	0.25***	0.25***	0.70***	0.24***
important to live in a city/place that one likes							0.16***	0.32***	0.17***
constant	2.79***	4.84***	3.06***	2.65***	4.46***	2.96***	2.12***	3.35***	2.39***
state dummies	yes	yes	yes	yes	yes	yes	yes	yes	yes
N	3707	3696	3722	3697	3686	3713	3688	3676	3703

+ p < 0.10,

* p < 0.05,

** p < 0.01,

*** p < 0.001;

robust std err

Effect sizes are consistent. Satisfaction with life as a whole and SWLS are both on scales 1-5, whereas life satisfaction ladder question is on scale 1-10, and correspondingly coefficients are about twice as large on the ladder question. In full specifications a3*, effect sizes on metro are about half of the coefficient on health, so in practical terms this means that living in a metro depresses one's happiness as much as going half way from fair health to poor health, for instance.

Next, we turn to SWLS components–regression results are in Table 4. In final five specifications b3*, the first two items, “life is close to ideal”, and “conditions of life excellent” are of similar magnitude at about .1. “Satisfied with life” in column b3d is insignificant.^4^ And two final items, “gotten the important things” and “would change almost nothing” are of greatest magnitude, especially the last one. Again, all the metro effect sizes are about 2x larger than simple mean differences from Table 2. Thus we find a broad support for our hypothesis that metros are less happy across multiple measures of SWB.

**Table 4:** OLS regressions of SWLS components.

	b2a life is close to ideal	b2b conditions of life excellent	b2c satisfied with life	b2d gotten the important things	b2e would change almost nothing	b3a life is close to ideal	b3b conditions of life excellent	b3c satisfied with life	b3d gotten the important things	b3e would change almost nothing
metro	-0.08+	-0.10*	-0.02	-0.12*	-0.16**	-0.11*	-0.12*	-0.04	-0.14**	-0.19**
age	-0.01	-0.01+	-0.01	-0.03***	-0.03**	-0.01	-0.02*	-0.01	-0.03***	-0.03**
age sq	0.00	0.00+	0.00	0.00***	0.00**	0.00+	0.00*	0.00	0.00***	0.00**
last year total family income	0.00***	0.00***	0.00***	0.00***	0.00***	0.00***	0.00***	0.00***	0.00***	0.00***
temp not working	-0.33	-0.39	-0.58	-0.13	-0.38	-0.30	-0.36	-0.55	-0.10	-0.34
unemployed	-0.33***	-0.28**	-0.29***	-0.39***	-0.33***	-0.31***	-0.26**	-0.27**	-0.37***	-0.31**
retired	0.07	0.11+	0.12*	0.20***	0.20**	0.06	0.10	0.11+	0.18**	0.18*
disabled	-0.22**	-0.23**	-0.23**	-0.23**	-0.25**	-0.21*	-0.23**	-0.22*	-0.23*	-0.24*
housekeeping	-0.21*	0.06	-0.07	0.07	0.01	-0.21*	0.06	-0.06	0.07	0.02
student	-0.16	-0.19	-0.16	-0.35+	-0.24	-0.17	-0.20	-0.17	-0.37+	-0.25
kids	-0.02	-0.05	-0.03	-0.00	-0.02	-0.02	-0.05	-0.03	-0.00	-0.02
college	-0.06	-0.04	-0.08*	-0.00	-0.16***	-0.07+	-0.05	-0.09*	-0.01	-0.17***
health	0.28***	0.32***	0.27***	0.20***	0.24***	0.27***	0.30***	0.26***	0.19***	0.22***
male	-0.06	-0.03	-0.11*	-0.18***	-0.13*	-0.04	-0.00	-0.09+	-0.15**	-0.11+
married	0.33***	0.28***	0.31***	0.38***	0.35***	0.33***	0.28***	0.30***	0.37***	0.35***
family unit size	0.02	0.03	0.04	0.03	0.04	0.02	0.03	0.04	0.04	0.04
black	0.11*	0.10*	0.19***	-0.01	0.17**	0.09*	0.08+	0.17***	-0.03	0.14*
other	0.11	0.11	0.15	0.12	0.14	0.11	0.11	0.15	0.12	0.14
asian	0.20	0.03	0.06	0.13	0.06	0.22	0.06	0.09	0.16	0.09
latino	0.32***	0.29***	0.29***	0.17*	0.20+	0.31***	0.28***	0.28***	0.16+	0.19+
important to live in a city/place that one likes						0.16***	0.19***	0.17***	0.16***	0.18***
constant	2.80***	2.69***	2.84***	3.34***	2.99***	2.30***	2.07***	2.27***	2.78***	2.38***
state dummies	yes	yes	yes	yes	yes	yes	yes	yes	yes	yes
N	3697	3692	3686	3691	3698	3687	3682	3676	3681	3688

+ p < 0.10,

* p < 0.05,

** p < 0.01,

*** p < 0.001;

robust std err

**Figure 2:**
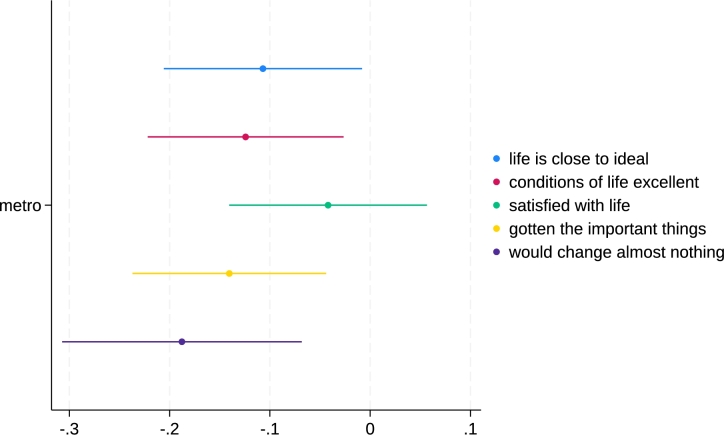
Coefficient plot of point estimates with 95% CI of models b3* from Table 4.

We finish with visualization of key “final” results from Table 4 in Fig. 2 showing full models b3*. It is clear that “would change almost nothing” is of greatest magnitude–urbanites would have changed things–they have regrets.

## 4 Conclusion and discussion

There are about a hundred studies on urban-rural happiness gradient, but all studies use a simple single-item measurement of SWB. Such limitation is understandable and common, as multi-item scale measurement is typically restricted to small-sample laboratory settings. And urbanicity deriving from place of residence by definition requires wide geographical coverage and large sample. This is the first study of urban-rural happiness gradient using elaborate multi-item scale measurement of SWB. Satisfaction With Life Scale (SWLS) confirms earlier single-item finding of urban-rural happiness gradient.

Effect sizes are about half of the coefficient on health, so in practical terms this means that living in a metro depresses one's happiness as much as going half way from fair health to poor health, for instance.

As compared to the first two items of SWLS scale “In most ways my life is close to my ideal,” and “The conditions of my life are excellent,” the largest difference is on the fifth item “If I could live my life over, I would change almost nothing” and almost as large on the fourth item, which has a similar meaning: “So far I have gotten the important things I want in life.”

Hence, a new finding is that urbanites fail especially on item “If I could live my life over, I would change almost nothing” indicating that urban way of life may result in regrets. Already 40 years ago, Campbell has noted that urbanites tend to find life frustrating an they think they weren't able to achieve their full share of happiness [8]. Furthermore, aspirations and comparisons are critical to explaining urban unhappiness [9]. Campbell's observations can help explain our study's results.

Arguably an urbanite has fuller life: more experience and achievement than rural folks–and more experience or achievement could perhaps result in more happiness. But urban life also increases expectations and aspirations, arguably more than experience or achievement.^5^ And while experience and achievement increase SWB, expectations and aspirations decrease it: SWB=experience+achievement−expectations−aspirations.

As city exposes one to multitude of stimuli and experiences [46], an urbanite is more likely to regret things in life and wish life went in a different direction, whereas in rural areas choices and pathways are more limited, constrained, and hence less regret-prone [55]. Perhaps, in a way, “ignorance is a bliss.” It remains for future research to explore it in detail.

There is an eye-opening book by a palliative nurse about the top regrets of the dying [62]. It is an useful resource for anyone interested in happiness–people on their deathbed have a full (lived their life) and honest (nothing to lose) perspective on what matters in life. None of the top regrets is about money, production, and consumption: “I wish I'd had the courage to live a life true to myself, not the life others expected of me,” “I wish I hadn't worked so hard,” “I wish I'd had the courage to express my feelings,” “I wish I had stayed in touch with my friends,” “I wish that I had let myself be happier.” If anything, is it actually production and consumption that result in regrets at the end of the life, as we devote our lives to them and little else. And capitalistic production and consumption “rat race” has its home in metros [52], [53], [54], [49], [29], [42].^6^

### 4.1 Limitations/future research

Data are observational, not experimental, and hence, causality may not be present. Urbanism, however, can only be studied using observational data–if readers have an idea for a quasi-experiment, please email me.

Crime and fear of crime are higher in cities and predict lower wellbeing–but we fail to control for them as we did not find any good measures in PSID. Thus, our results are likely stronger than they should have been controlling for crime. Still, even controlling for crime, there is urban penalty in life satisfaction [45], and so likely in other wellbeing measures as well. Furthermore, crime is an integral part of urbanism, at least in the US–the larger the place the more crime [3]. Still, future research should take into account crime and fear of crime.

While results are likely to generalize to other developed countries, in the poorest countries, such as sub-Saharan Africa, the relationship may not hold or even reverse. In the very poorest places urbanism is likely to be associated with greater wellbeing as rural areas often lack necessities such as clean water or adequate shelter. In terms of demographics, we study adults–future research can study children and elderly–these sub-populations are likely to be even less happy in cities than adults as cities are in general build for adults to work and consume.

We have used here 8 measures of wellbeing: life satisfaction, Cantril ladder, SWLS, and each of the 5 components of SWLS. It is a great improvement over vast majority of existing studies using only 1 measure, typically life satisfaction. Still, future research can go further in this direction and use even more measures. One fruitful direction could be to measure better urban regrets.

Initial findings of lower life satisfaction in cities have been recently enriched by investigations into interactions, i.e., some groups are less happy than others in cities [24], [32], [47], [11], [10], [25]. A similar direction can be taken with findings from present study.

## CRediT authorship contribution statement

**Adam Okulicz-Kozaryn:** Writing – review & editing, Writing – original draft, Visualization, Validation, Software, Methodology, Investigation, Formal analysis, Data curation, Conceptualization.

## Declaration of Competing Interest

There is no conflict of interest.

## Data Availability

Data are public: https://psidonline.isr.umich.edu/. Code available from the author upon request.

## Appendix A Supplementary material

MMC Variables' definitions, coding, and distributions; cross-correlations; and robustness check: additional results. MMC

## Footnotes

2 Burger et al. [7] also uses faulty Gallup data as elaborated in Okulicz-Kozaryn and Valente [48]–in general, one should steer away from Gallup happiness data–Gallup charges $30,000 for access (per one year), clearly “happiness industry”, not happiness research Davies [12].

3 Results on racial categories are unexpected. Blacks and Latinos are happier than whites, and we do not have an explanation for that. Except perhaps that minorities have advanced recently socio-economically as compared to whites in the US. Race is not a topic of inquiry here, but a statistical control only.

4 Note, wording of this question is different from general life satisfaction question in Table 3.

5 For instance people tend to make upward comparisons [21] and hence end up relatively deprived and there are vastly more comparisons to be made in a city. It is better to be a small fish in a small pond than even a quite large fish in a very large pond.

6 There are multiple advantages to urbanism, notably emancipative [61], [63], environmental [28], and creative [19]. Yet, in rural areas, too, one can be creative [37], [20], free, and environmentally sustainable [60], [59].
