# Supplementary material for: Urban regrets (Unhappy metros: Satisfaction with life scale (SWLS))
Source: Heliyon. 2024 May 13;10(11):e30729. doi: 10.1016/j.heliyon.2024.e30729 (PMC11214393; doi:10.1016/j.heliyon.2024.e30729)
Supplement: MMC — Variables' definitions, coding, and distributions; cross-correlations; and robustness check: additional results. [file mmc1.pdf]

Online Appendix:

Urban Regrets
(Unhappy Metros: Satisfaction With Life Scale (SWLS))

Friday 19th April, 2024 07:38

Pro-Urban Bias in Economics

Despite the evidence, some economists are still trying to argue that happiness has its home in the city. The pro-urban bias in economics is arguably due to economic theory. In economics, *happiness* ≈ *utility* ≈ *money*–there is most money in cities, there is most utility, and there must be most happiness as well, economic thinking goes. Economists cherry-pick data, e.g., the poorest African countries where indeed urbanites are happier, to find "the evidence" to support the economic theory (Glaeser 2011, Glaeser et al. 2016, Burger et al. 2020).

Variables' definitions, coding, and distributions

Metro definition

The metro v non-metro classification is based on the following:

metro	beale rural-urban code	description
1	1	Metro: Counties in metro areas of 1 million population or more
1	2	Metro: Counties in metro areas of 250,000 to 1 million population
1	3	Metro: Counties in metro areas of fewer than 250,000 population
0	4	Nonmetro: Urban population of 20,000 or more, adjacent to a metro area
0	5	Nonmetro: Urban population of 20,000 or more, not adjacent to a metro area
0	6	Nonmetro: Urban population of 2,500 to 19,999, adjacent to a metro area
0	7	Nonmetro: Urban population of 2,500 to 19,999, not adjacent to a metro area
0	8	Nonmetro: Completely rural or less than 2,500 urban population, adjacent to a metro area
0	9	Nonmetro: Completely rural or less than 2,500 urban population, not adjacent to a metro area

Table 1: metro variable: Metropolitan/Non-metropolitan Indicator: This indicator is derived from the 2013 Beale-Ross Rural-Urban Continuum Codes published by USDA based on matches to the FIPS state and county codes: 1. Metropolitan area (Beale-Ross Code ER775923= 1-3); 0. Non-metropolitan area (Beale-Ross Code ER775923= 4-9). Each county in the U.S. is assigned one of the 9 codes.

The PSID 2015 family file codebook (`https://psidonline.isr.umich.edu/documents/psid/codebook/fam2015er_codebook.pdf`) defines the BEALE RURAL-URBAN CODE:

> ER65453 "BEALE RURAL-URBAN CODE" NUM(2.0) Metropolitan/Non-metropolitan Indicator 2013 Beale-Ross Rural-Urban Continuum Code for 2015 Residence
>
> This variable is suppressed (filled with zeroes) in the public release file to protect the anonymity of respondents. The data are available in a separate file: FAM19YEAR_rst where Year is the corresponding Family

File year (i.e. FAM1968_rst contains data for suppressed variables from the 1968 file). This file is available to qualified users under special contractual arrangements with the PSID. For more information, contact us at PSIDhelp@umich.edu and request County Level Identifiers restricted file. These codes are based on matches to the FIPS state and county codes against the 2013 Rural-Urban Continuum Codes published by USDA downloaded from https://www.ers.usda.gov/data-products/rural-urban-continuum-codes/.aspx The 2013 Rural-Urban Continuum Codes form a classification scheme that distinguishes metropolitan counties by the population size of their metro area, and nonmetropolitan counties by degree of urbanization and adjacency to a metro area. The official Office of Management and Budget (OMB) metro and nonmetro categories have been subdivided into three metro and six nonmetro categories. Each county in the U.S. is assigned one of the 9 codes. This scheme allows researchers to break county data into finer residential groups, beyond metro and nonmetro, particularly for the analysis of trends in nonmetro areas that are related to population density and metro influence. The Rural-Urban Continuum Codes were originally developed in 1974. They have been updated each decennial since (1983, 1993, 2003, 2013), and slightly revised in 1988. Note that the 2013 Rural-Urban Continuum Codes are not directly comparable with the codes prior to 2000 because of the new methodology used in developing the 2000 metropolitan areas.

Variables' coding, and distributions

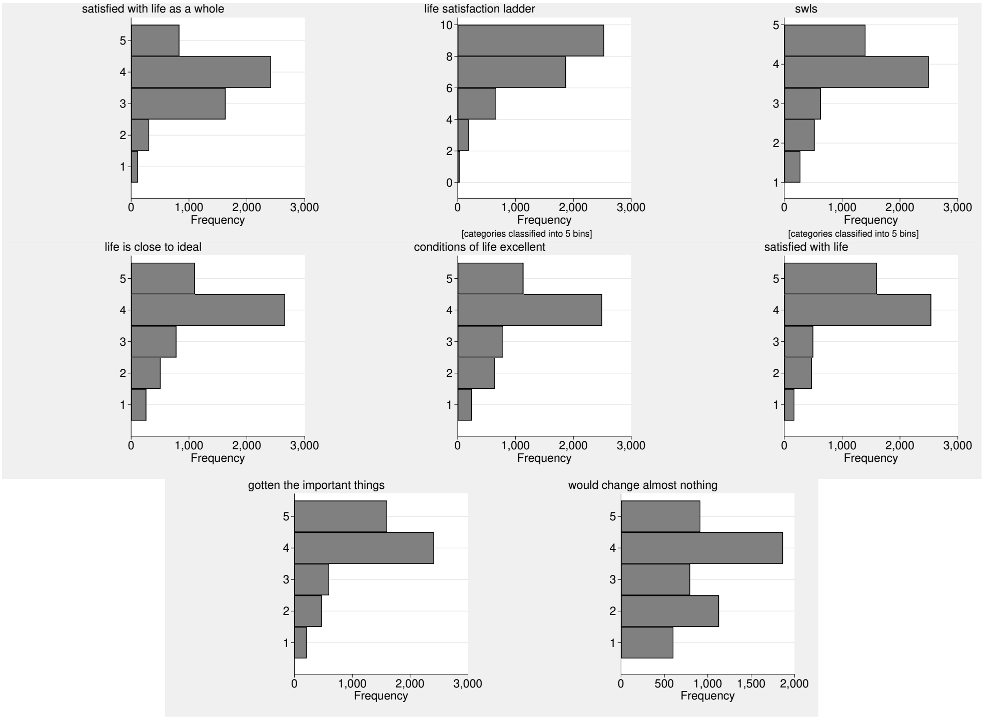

Figure 1: Variables' distribution.

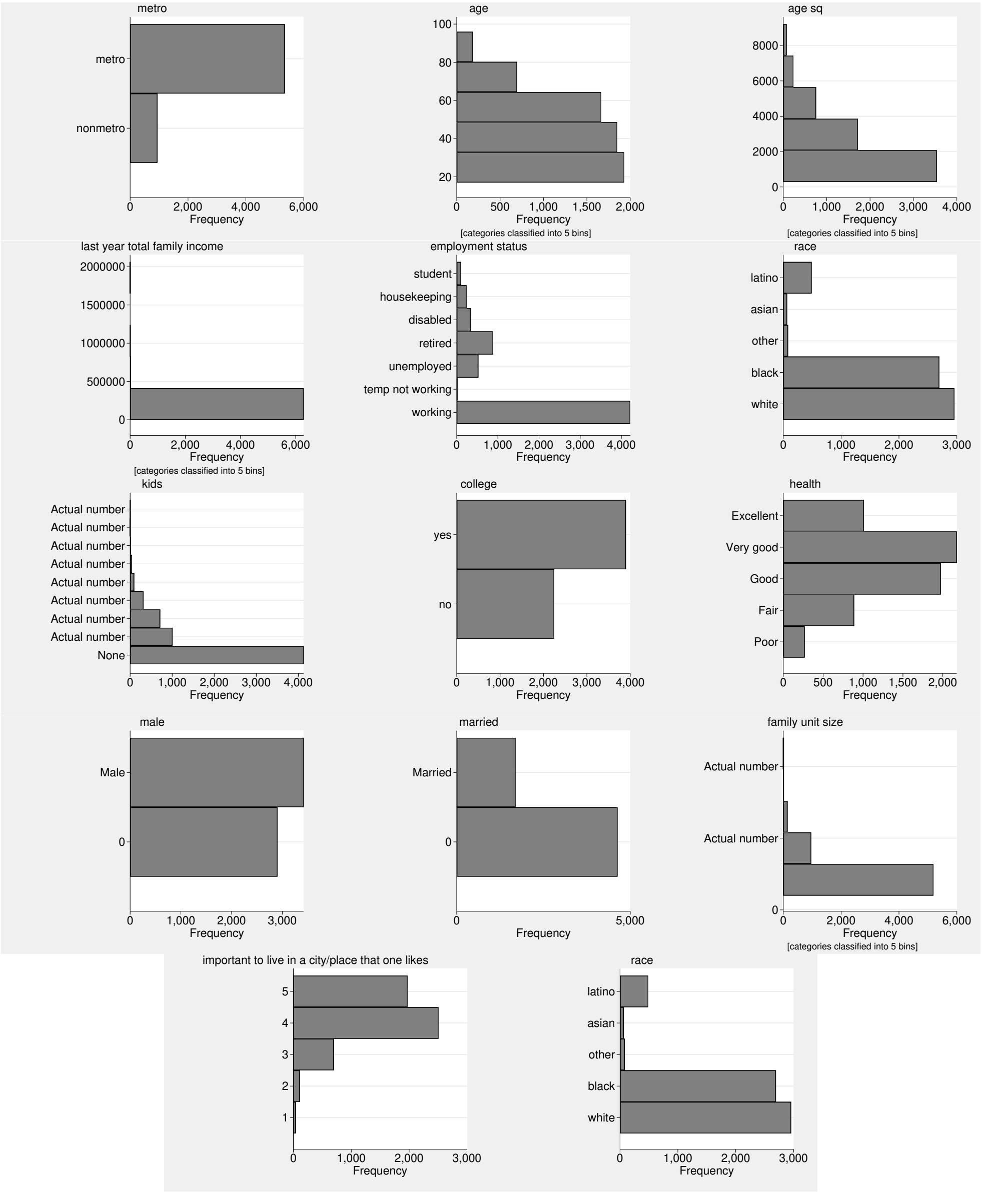

Figure 2: Variables' distribution.

1 Cross-correlations and collinearity/VIF

The three global SWB measures correlate considerably at about .75.

```
 pwcorr WB16A1 WB16A2 swls

             |   WB16A1   WB16A2     swls
-------------+---------------------------
      WB16A1 |   1.0000
      WB16A2 |   0.7579   1.0000
        swls |   0.7165   0.7420   1.0000
```

Next we turn to pairwise correlations of swls items. in general they are high at about .7-.9, except the last two items and especially the last item that are lower at .5-.6.

```
. pwcorr swls WB16A3A WB16A3B WB16A3C WB16A3D WB16A3E
```

	swls	WB16A3A	WB16A3B	WB16A3C	WB16A3D	WB16A3E
swls	1.0000					
WB16A3A	0.8586	1.0000				
WB16A3B	0.8675	0.7477	1.0000			
WB16A3C	0.8694	0.7267	0.7513	1.0000		
WB16A3D	0.8049	0.6058	0.6042	0.6456	1.0000	
WB16A3E	0.7707	0.5281	0.5361	0.5375	0.5230	1.0000

We finish pairwise correlations by look at right hand side variables. The first column, metro has weak correlations with other variables at below .2. In general correlations are weak.

```
. pwcorr met age age2  inc emp rac  kid col hea male mar nFU   WB16A4B  rac
```

	met	age	age2	inc	emp	rac	kid
met	1.0000						
age	-0.0360	1.0000					
age2	-0.0393	0.9838	1.0000				
inc	0.0510	0.1082	0.0767	1.0000			
emp	-0.0337	0.3580	0.3847	-0.2009	1.0000		
rac	0.1424	-0.1105	-0.1114	-0.1407	-0.0185	1.0000	
kid	0.0077	-0.2710	-0.2865	0.0250	-0.0901	0.1039	1.0000
col	0.0966	-0.0567	-0.0650	0.2425	-0.1704	-0.0822	-0.0452
hea	0.0279	-0.1888	-0.1815	0.1901	-0.2959	-0.0458	0.0339
male	-0.0353	-0.0232	-0.0336	0.2746	-0.1083	-0.0908	-0.0810
mar	-0.0252	0.1739	0.1480	0.4323	-0.0828	-0.0923	0.1081
nFU	0.0118	-0.1457	-0.1749	0.1695	-0.0943	0.0923	0.8555
WB16A4B	0.0905	-0.0210	-0.0209	0.0393	-0.0446	0.0332	0.0053
rac	0.1424	-0.1105	-0.1114	-0.1407	-0.0185	1.0000	0.1039

	col	hea	male	mar	nFU	WB16A4B	rac
col	1.0000						
hea	0.1346	1.0000					
male	0.0411	0.1154	1.0000				
mar	0.1300	0.1142	0.5237	1.0000			
nFU	-0.0332	0.0396	0.0775	0.3530	1.0000		
WB16A4B	0.0615	0.1037	-0.0425	-0.0034	0.0002	1.0000	
rac	-0.0822	-0.0458	-0.0908	-0.0923	0.0923	0.0332	1.0000

Below we repeat full model b3e and run VIF statistics for it (other full models b3* are similar in terms of VIF). VIF on all variables is small, < 5, except age and age^2, as expected. Also size of family unit and number of children correlate strongly at .85, but that is far from being close to perfect correlation. And one state dummy has VIF slightly above 5. We conclude that there are no problems with collinearity.

```
 reg WB16A3E met age age2  inc E2-E7  kid col hea male mar nFU i.sFIPS R2-R5
> WB16A4B , robust

Linear regression                               Number of obs     =      3,688
                                                F(68, 3617)       =          .
                                                Prob > F          =          .
                                                R-squared         =     0.1131
                                                Root MSE          =     1.2332
```

WB16A3E	Coefficient	Robust std. err.	t	P>|t|	[95% conf.	interval]
met	-0.19	0.06	-3.08	0.00	-0.31	-0.07
age	-0.03	0.01	-2.82	0.00	-0.05	-0.01
age2	0.00	0.00	3.27	0.00	0.00	0.00

inc	0.00	0.00	3.76	0.00	0.00	0.00
E2	-0.34	0.38	-0.90	0.37	-1.09	0.40
E3	-0.31	0.10	-3.14	0.00	-0.50	-0.12
E4	0.18	0.07	2.50	0.01	0.04	0.33
E5	-0.24	0.10	-2.49	0.01	-0.43	-0.05
E6	0.02	0.12	0.14	0.89	-0.22	0.25
E7	-0.25	0.23	-1.11	0.27	-0.71	0.20
kid	-0.02	0.04	-0.54	0.59	-0.11	0.06
col	-0.17	0.05	-3.55	0.00	-0.26	-0.08
hea	0.22	0.02	10.08	0.00	0.18	0.27
male	-0.11	0.06	-1.93	0.05	-0.22	0.00
mar	0.35	0.07	5.21	0.00	0.22	0.48
nFU	0.04	0.04	1.15	0.25	-0.03	0.11
sFIPS						
Actual s..)	0.03	0.28	0.11	0.91	-0.51	0.57
Actual s..)	-0.20	0.26	-0.79	0.43	-0.71	0.31
Actual s..)	0.15	0.23	0.65	0.52	-0.30	0.59
Actual s..)	-0.12	0.19	-0.62	0.53	-0.50	0.26
Actual s..)	-0.37	0.24	-1.53	0.13	-0.84	0.10
Actual s..)	0.01	0.28	0.04	0.97	-0.53	0.55
Actual s..)	-0.86	0.68	-1.26	0.21	-2.19	0.47
Actual s..)	-0.27	0.37	-0.74	0.46	-1.00	0.45
Actual s..)	-0.18	0.20	-0.88	0.38	-0.58	0.22
Actual s..)	-0.08	0.21	-0.41	0.68	-0.49	0.32
Actual s..)	-0.55	0.23	-2.35	0.02	-1.01	-0.09
Actual s..)	-0.47	0.39	-1.19	0.24	-1.24	0.30
Actual s..)	-0.06	0.21	-0.27	0.79	-0.47	0.35
Actual s..)	-0.03	0.21	-0.16	0.87	-0.45	0.39
Actual s..)	-0.09	0.22	-0.40	0.69	-0.53	0.35
Actual s..)	-0.23	0.31	-0.74	0.46	-0.83	0.38
Actual s..)	-0.57	0.25	-2.23	0.03	-1.06	-0.07
Actual s..)	0.01	0.24	0.06	0.95	-0.46	0.49
Actual s..)	-0.18	0.32	-0.55	0.58	-0.81	0.46
Actual s..)	-0.13	0.21	-0.61	0.54	-0.55	0.29
Actual s..)	0.04	0.24	0.15	0.88	-0.44	0.51
Actual s..)	-0.16	0.20	-0.82	0.41	-0.55	0.23
Actual s..)	-0.30	0.23	-1.31	0.19	-0.75	0.15
Actual s..)	0.00	0.20	0.01	0.99	-0.39	0.40
Actual s..)	-0.41	0.22	-1.86	0.06	-0.83	0.02
Actual s..)	-0.39	0.54	-0.72	0.47	-1.46	0.67
Actual s..)	-0.11	0.28	-0.40	0.69	-0.66	0.44
Actual s..)	-0.30	0.35	-0.87	0.38	-0.98	0.38
Actual s..)	0.25	0.57	0.43	0.66	-0.86	1.35
Actual s..)	-0.03	0.23	-0.14	0.89	-0.47	0.41
Actual s..)	0.19	0.36	0.52	0.60	-0.52	0.90
Actual s..)	-0.40	0.21	-1.94	0.05	-0.80	0.00
Actual s..)	-0.15	0.20	-0.78	0.43	-0.54	0.23
Actual s..)	-0.20	0.20	-0.98	0.32	-0.58	0.19
Actual s..)	0.01	0.30	0.03	0.97	-0.59	0.61
Actual s..)	-0.31	0.23	-1.39	0.17	-0.76	0.13
Actual s..)	-0.16	0.21	-0.80	0.42	-0.57	0.24
Actual s..)	1.21	0.22	5.49	0.00	0.78	1.65
Actual s..)	0.00	0.20	0.02	0.99	-0.39	0.40
Actual s..)	0.44	0.33	1.33	0.18	-0.21	1.08
Actual s..)	0.05	0.23	0.20	0.84	-0.40	0.49
Actual s..)	-0.25	0.20	-1.28	0.20	-0.64	0.13
Actual s..)	-0.16	0.28	-0.55	0.58	-0.71	0.40
Actual s..)	-0.57	0.68	-0.84	0.40	-1.91	0.76
Actual s..)	-0.29	0.21	-1.35	0.18	-0.70	0.13
Actual s..)	-0.08	0.23	-0.33	0.74	-0.53	0.38
Actual s..)	0.19	0.64	0.29	0.77	-1.06	1.44
Actual s..)	-0.17	0.28	-0.60	0.55	-0.72	0.38
Actual s..)	-0.67	0.61	-1.11	0.27	-1.86	0.52
R2	0.14	0.06	2.55	0.01	0.03	0.25
R3	0.14	0.20	0.71	0.48	-0.24	0.52
R4	0.09	0.20	0.45	0.65	-0.30	0.48
R5	0.19	0.11	1.65	0.10	-0.04	0.41
WB16A4B	0.18	0.03	6.61	0.00	0.13	0.24
_cons	2.38	0.34	7.07	0.00	1.72	3.04

```
------------------------------------------------------------------------------

.
end of do-file

. estat vif

    Variable |       VIF       1/VIF  
-------------+----------------------
         met |      1.26    0.795907
         age |     51.09    0.019573
        age2 |     53.11    0.018828
         inc |      1.46    0.684749
          E2 |      1.01    0.987233
          E3 |      1.12    0.895763
          E4 |      2.12    0.472227
          E5 |      1.23    0.810138
          E6 |      1.12    0.894316
          E7 |      1.03    0.968434
         kid |      5.19    0.192577
         col |      1.21    0.829258
         hea |      1.24    0.803982
        male |      1.75    0.571620
         mar |      2.37    0.422665
         nFU |      5.51    0.181648
       sFIPS |
          2  |      1.13    0.884454
          4  |      1.72    0.579965
          5  |      2.16    0.462856
          6  |      5.39    0.185638
          8  |      1.83    0.547489
          9  |      1.41    0.711340
         10  |      1.07    0.937747
         11  |      1.34    0.748052
         12  |      3.35    0.298285
         13  |      3.03    0.329942
         15  |      1.03    0.970215
         16  |      1.12    0.893408
         17  |      3.00    0.333742
         18  |      2.89    0.345894
         19  |      2.07    0.483980
         20  |      1.40    0.715007
         21  |      1.79    0.558552
         22  |      1.80    0.555262
         23  |      1.17    0.857544
         24  |      3.01    0.332357
         25  |      2.00    0.500206
         26  |      3.80    0.263338
         27  |      2.10    0.477202
         28  |      3.29    0.304203
         29  |      2.61    0.382483
         30  |      1.11    0.904496
         31  |      1.36    0.735391
         32  |      1.39    0.717198
         33  |      1.07    0.933794
         34  |      2.31    0.433831
         35  |      1.09    0.921561
         36  |      3.24    0.308816
         37  |      4.17    0.239916
         39  |      3.65    0.274162
         40  |      1.31    0.761926
         41  |      2.17    0.461387
         42  |      3.45    0.289588
         44  |      1.03    0.974856
         45  |      3.44    0.290882
         46  |      1.22    0.818879
         47  |      2.14    0.467248
         48  |      4.31    0.231835
         49  |      1.58    0.632584
         50  |      1.07    0.935832
         51  |      2.71    0.368869
```

```
         53  |      2.17    0.461877
         54  |      1.09    0.918095
         55  |      1.64    0.608558
         56  |      1.07    0.937203
          R2 |      1.74    0.574707
          R3 |      1.03    0.972673
          R4 |      1.04    0.963538
          R5 |      1.15    0.869545
     WB16A4B |      1.05    0.955985
-------------+----------------------
    Mean VIF |      3.47
```

Robustness Check: Additional Results

Repeating models a3a a3b a3c and b3a-b3e, but with religiosity, city satisfaction, and industry dummies. Conclusion: results substantively very similar.

Table 2: OLS regressions of SWB.

	c3a satisfied with life as a whole	c3b life satisfaction ladder	c3c swls	d3a	d3b	d3c	d3d	d3e
metro	-0.14***	-0.23**	-0.10**	-0.09+	-0.12*	-0.03	-0.12*	-0.14*
age	-0.01	-0.00	-0.02*	-0.01	-0.02*	-0.01	-0.03***	-0.02*
age sq	0.00	0.00	0.00*	0.00	0.00	0.00	0.00***	0.00*
last year total family income	0.00**	0.00***	0.00***	0.00***	0.00***	0.00***	0.00***	0.00***
temp not working	-0.25	-0.72	-0.43	-0.39	-0.44	-0.65	-0.21	-0.39
unemployed	-0.17*	-0.42**	-0.29***	-0.32***	-0.25**	-0.24**	-0.35***	-0.35***
retired	0.09	-0.02	0.04	-0.07	0.01	0.03	0.15*	0.00
disabled	-0.13+	-0.45**	-0.33***	-0.36***	-0.34***	-0.32**	-0.28**	-0.45***
housekeeping	-0.03	-0.11	-0.04	-0.26*	0.05	-0.05	0.11	-0.09
student	-0.16	-0.40	-0.21	-0.15	-0.18	-0.13	-0.33+	-0.27
kids	-0.04	-0.05	-0.00	-0.00	-0.03	-0.00	0.03	-0.01
college	-0.05	-0.17*	-0.07*	-0.05	-0.04	-0.09*	0.00	-0.15**
health	0.23***	0.47***	0.20***	0.23***	0.26***	0.21***	0.14***	0.17***
male	-0.03	-0.02	-0.04	0.01	0.03	-0.03	-0.11*	-0.10
married	0.17***	0.46***	0.28***	0.28***	0.24***	0.27***	0.32***	0.32***
family unit size	0.05+	0.03	0.01	0.01	0.01	0.02	0.01	0.02
black	0.17***	0.46***	0.08*	0.07	0.07	0.16***	-0.01	0.12*
other	0.32*	0.57*	0.19	0.19	0.18	0.25	0.18	0.17
asian	0.20	0.31	0.18	0.30+	0.12	0.12	0.22	0.13
latino	0.27***	0.72***	0.25***	0.34***	0.28***	0.30***	0.14	0.23*
important to live in a city/place that one likes	0.06**	0.13**	0.06**	0.05*	0.07**	0.06*	0.06*	0.06*
A4J HOW IMPORTANT STRONG RELIGIOUS FAITH	-0.04***	-0.08**	-0.04***	-0.05***	-0.05***	-0.04**	-0.03*	-0.06***
A5B HOW SATISFIED W/ CITY	0.29***	0.58***	0.32***	0.30***	0.33***	0.32***	0.29***	0.34***
constant	1.98***	2.95***	2.17***	2.12***	1.88***	2.05***	2.49***	2.23***
industry dummies	yes	yes	yes	yes	yes	yes	yes	yes
state dummies	yes	yes	yes	yes	yes	yes	yes	yes
N	3658	3646	3672	3656	3651	3647	3650	3657

+ p<0.10, * p<0.05, ** p<0.01, *** p<0.001; robust std err

References

Burger, M. J., P. S. Morrison, M. Hendriks, and M. M. Hoogerbrugge (2020): "Urban-Rural Happiness Differentials across the World," *World Happiness Report*.

Glaeser, E. (2011): *Triumph of the City: How Our Greatest Invention Makes Us Richer, Smarter, Greener, Healthier, and Happier*, Penguin Press, New York NY.

Glaeser, E. L., J. D. Gottlieb, and O. Ziv (2016): "Unhappy Cities," *Journal of Labor Economics*, 34, S129–S182.
